# Parents’ and adolescents’ perception of traffic- and crime-related safety as correlates of independent mobility among Belgian adolescents

**DOI:** 10.1371/journal.pone.0204454

**Published:** 2018-09-28

**Authors:** Francisco Javier Huertas-Delgado, Lieze Mertens, Palma Chillon, Delfien Van Dyck

**Affiliations:** 1 PA-Help "Physical Activity for Health Promotion" research group, Teaching Centre La Inmaculada, University of Granada, Granada, Spain; 2 Ghent University, Faculty of Medicine and Health Sciences, Department of Movement and Sports Sciences, Gent, Belgium; 3 Research Foundation Flanders, Brussels, Belgium; 4 PROFITH “Promoting FITness and Health through physical activity” research group, Department of Physical Education and Sport, Faculty of Sport Sciences, University of Granada, Granada, Spain; Western University, CANADA

## Abstract

The independent mobility (IM), defined as the freedom of young people to travel without adult supervision, has been related to the physical activity time, the acquisition of personal autonomy, to less intense fear of crime, and to a stronger feeling of being part of their community and other health and social benefits. The aims of this study were to compare parents’ and adolescents’ traffic- and crime-related safety perceptions of their neighborhood and to analyze the associations of these perceptions with adolescents’ IM. A total of 291 adolescents and their parents completed the Neighborhood Environment Walkability Scale (NEWS) questionnaire. Multilevel (two-level models: individual level—neighborhood level) regression analyses were conducted to examine whether the environmental perceptions differed between parents and adolescents and the association between the parental and adolescents’ perception to the IM and the active independent mobility (AIM). Parents reported a more negative perception of traffic (except for amount and speed) and crime-related safety. Adolescents’ environmental perceptions were not associated with their IM but parental perceptions of traffic- and crime-related safety were associated with IM and with active IM, although not all associations were in the expected direction. Future urban policy efforts should address environments where parents perceive sufficient levels of safety to increase the levels of IM in adolescents.

## Background

The World Health Organization (WHO) recommends at least 60 minutes of moderate-to-vigorous physical activity (PA) per day in order to achieve health benefits in youth aged 5 to 17 years old [1]. These benefits include a better mental health status, better self-esteem and physiological well-being [2], improved academic achievement [3], better cardiorespiratory fitness levels [4], and lower rates of overweight and obesity [5]. Nevertheless, PA levels decline as children become adolescents [6]. Furthermore, as young people with high PA levels are more likely to become active adults [7], promoting daily PA among youth has been identified as a global health priority [1].

Active living is a broad concept that incorporates four domains of PA: active recreation, household activities, active transport, and occupation activities [8]. Active transport, which includes walking and cycling as means of transport [9], is a potential source to reach sufficient levels of moderate PA for adolescents [10,11] and can be easily integrated in their daily routine [12]. Furthermore, it has been proven that walking and cycling as forms of transport in youth are associated with other health benefits such as an improved body composition and higher cardiorespiratory fitness [13].

Independent mobility (IM) is defined as the freedom of young people to travel without adult supervision [14]. IM has been positively related to the amount of active commuting to school, to the total daily PA [15], and specifically to non-school-based PA [16]. IM is related to the acquisition of personal autonomy [17], to less intense fear of crime, and to a stronger feeling of being part of their community [18]. In addition, IM is also important to promote adolescents’ social, cognitive, and emotional development [19], which are closely related to the imbalance suffered by youth in this age period [20]. Despite all the previous benefits, IM has declined in recent decades primarily due to traffic danger and fear of assault in different countries such as Australia, Norway, Denmark, England, and Germany [19,21,22].

In the ecological framework proposed by Mandic and colleagues, the adolescents’ mode of commuting is influenced by personal, social, and environmental correlates [23]. These three levels of correlates may influence IM in adolescents. For example, boys are granted with more IM [24]. Furthermore, children’s skills [25] and family socioeconomic status [26] are related to lower levels of IM and physical activity [27]. Previous studies showed that longer distances, heavy traffic and perceiving the street as unsafe are related to lower IM in adolescents [28–30] and, on the other hand, higher social connection is related to higher IM [31]. Moreover, a Canadian study showed that the main reasons for parents to escort youth to school were fear of strangers and crime, as well as traffic volume outside schools [30]. On the one hand, parental perceptions of traffic danger [32] and unsafe environment [33] seem to be important to determine IM, and, on the other hand, adolescents’ own perceptions of safety can determine their activities in their free time (e.g. the mode of commuting or leisure-time physical activity) [34]. Consequently, creating safe neighborhoods to improve their perception in adolescents could increase IM [25].

Parents determine the distance that children are allowed to travel without supervision [35] and they can restrict the IM licenses due to unsafe environments [34]. At younger ages, travel choice may be more influenced by traffic and safety concerns of parents, but when children grow, they become more involved in transport-related decisions, and their opinion may be more important [36]. Adolescence (12–16 years old) is the period in which youths make the transition into and out the secondary high school [37]. The start of adolescence (12 years old) usually coincides with the change from primary to secondary school and results in a change of the route to school, new friendships, and involvement in different activities that promote greater independence [38], thus adolescents become more autonomous.

Currently, it is unclear which has more weight in the decision of IM, whether parental or adolescents’ perceptions. Some previous studies examined the relation between adolescents’ and parents’ neighborhood perceptions but none examined their specific association with IM. For instance, Carver et al. reported that children’s and adolescents’ perceptions of safety were higher than the parental perception, and were more strongly related to active commuting in the neighborhood [39] and Schoeppe et al. related higher rates of IM when parents had a higher educational level and reported higher social connection [40]. Systematic reviews about IM assessed the methodological approach [41] or its relation to physical activity and weight status [15]. To the best of our knowledge, the relation between parents’ and adolescents’ perception of traffic- and crime-related safety and their association with IM has not been studied before. Addressing whose perceptions (adolescents or parents) are more relevant to determine IM is important because it would allow researchers and practitioners to prepare effective interventions to increase IM and, indirectly, to increase the rates of active transportation [42].

The purpose of this study was two-fold: the first aim was to compare parental and adolescents’ traffic- and crime-related safety perceptions of the same neighborhood environment. The second aim was to analyze the association of parental and adolescents’ neighborhood-related traffic and crime safety perceptions with adolescents’ IM.

## Methods

### Protocol and procedures

The data were collected between September 2014 and March 2016 as part of the follow-up measurements of the Belgian Environmental Physical Activity Study in children (BEPAS-child) [43] that took place between December 2011 and May 2013. The study was conducted in Ghent, Belgium. The participants were nested within neighborhoods (n = 98). The number of participants per neighborhood ranged from 1 to 13 and the included neighborhoods were spread across the city and suburbs (i.e. low and high walkable areas) in Ghent, and across low and high SES areas. The participants first received an information letter, informing that a researcher would visit them at home a few days later and introducing the purpose of the follow-up study. Of the 606 children who participated in BEPAS-child, 375 adolescents agreed to participate in this follow-up study and completed a survey about their PA and perceptions of their neighborhood environment. The study was limited to 12–15 years old because it corresponded to the follow-up of a study conducted three years later with children. Therefore the sample accessible to this work was young adolescents. After selecting those who provided complete data, the final sample was composed of 291 adolescents and their parents. Ethical approval (i.e. sample, procedure, consents (active and opt-out), instruments) was obtained from the Ethics Committee of Ghent University, and participants’ informed consent was obtained prior to the data collection. The adolescents provided active consent (adolescents signed the informed consent if they want to participate) and the parents provided passive consent a letter was delivered to all the parents and they could retract permission returning the document signed).

### Measures

#### Socio-demographic characteristics

The adolescents self-reported their age, gender, height, and weight, and their parents self-reported their age, gender, height, weight, and highest educational level. The educational level of the parents was categorized into "low education" (no education, primary, lower secondary, or higher secondary) or "high education" (bachelor or master degree).

#### Perceived neighborhood environmental factors

The Neighborhood Environment Walkability Scale for Youth (NEWS-Y) and its version for adults [44] were used to assess parents’ and adolescents’ perception of the neighborhood environment. For this study, the 8 items of traffic safety and the 6 items of the safety from crime subscales were used, since these are common items asked exactly in the same way to both parents and adolescents (Table 1). All items were answered on a four-point Likert scale (from 1 = strongly disagree to 4 = strongly agree). The reliability of the questionnaire is acceptable for parents (ICC between 0.61–0.78) and for adolescents (ICC between 0.56–0.87). The internal consistency of the two subscales was acceptable for all participant groups (α between 0.72 and 0.90) [44].

**Table 1:** Perceived traffic safety and crime-related safety items.

NEWS	NEWS-Y
**Traffic safety**	**Traffic safety**
1. There is so much traffic in nearby streets that it’s hard or unpleasant for my child to walk in my neighborhood (alone or with someone else).	1. There is so much traffic in nearby streets that it’s hard or unpleasant to walk in my neighborhood (alone or with someone else).
2. In the nearby streets traffic usually drives slowly (50km/hour or less).	2. In the nearby streets traffic usually drives slowly (50km/hour or less).
3. Most drivers drive faster than the maximum speed limit in our neighborhood.	3. Most drivers drive faster than the maximum speed limit in my neighborhood.
4. There are a lot of exhaust fumes when I walk in my neighborhood.	4. There are a lot of exhaust fumes when I walk in my neighborhood.
5. At night the streets are well-lit in our neighborhood.	5. At night the streets are well-lit in my neighborhood.
6. In the streets in our neighborhood pedestrians and cyclists are very visible to people from their houses.	6. In the streets in my neighborhood pedestrians and cyclists are very visible to people from their houses.
7. There are zebra crossings and traffic lights in our neighborhood to help pedestrians at busy places.	7. There are zebra crossings and traffic lights in my neighborhood to help pedestrians at busy places.
8. I think it is safe to allow my child to cross the road in our neighborhood.	8. I feel safe crossing the roads in my neighborhood.
**Crime-related safety**	**Crime-related safety**
9. There is a lot of crime in our neighborhood.	9. There is a lot of crime in my neighborhood
10. Because of the high level of crime in our neighborhood it’s not safe for my child to walk at night (alone or with someone else).	10. Because of the high level of crime in my neighborhood it’s not safe to walk at night (alone or with someone else).
11. Allowing my child to play outside in the neighborhood (e.g. in the garden or on the street) worries me because I’m afraid that my child might be bothered by a stranger.	11. I am worried about being outside on my own in the neighborhood (e.g. in the garden or on the street) because I am frightened of being bothered by a stranger.
12. Allowing my child to play with friends in the neighborhood worries me because I’m afraid that my child might be bothered by a stranger.	12. I am worried about being with friends in the neighborhood because I am frightened of being bothered by a stranger.
13. Allowing my child to play or walk on the street and in my neighborhood alone or with friends worries me because I’m afraid that my child might be bothered by a stranger.	13. I am worried about being outside or being on the street on my own or with friends in my neighborhood because I am frightened of being bothered by a stranger.
14. Allowing my child to be alone or with friends in a nearby park worries me because I’m afraid that my child might be bothered by a stranger.	14. I am worried about being in a nearby park on my own or with friends because I am frightened of being bothered by a stranger.

#### Independent mobility (IM)

Adolescents’ IM is commonly assessed by means of a questionnaire [41]. In the current study, we asked adolescents about the average time they spent travelling without accompaniment per trip in a usual week, separately for (i) walking, (ii) cycling, and (iii) using public transport in order to assess adolescents’ IM. Only 15% of the participants reported 0 minutes of IM in all modes of commuting. Therefore, the mean of the time walking, cycling, and using public transport without adult accompaniment was used to define the variable “IM”. The variable “active IM (AIM)” was determined by the mean of the time using active modes of commuting (i.e. walking and cycling) without adult accompaniment.

### Statistical analyses

Descriptive statistics were reported for all relevant variables. Multilevel (two-level models: individual level—neighborhood level) regression analyses were conducted to examine whether the environmental perceptions differed between parents and adolescents. The age group (parents versus adolescents) was included as an independent variable and the perceptions -the 14 items of traffic safety and crime safety- were alternately entered as dependent variables in the regression models. All analyses were controlled for adolescents’ gender and parents’ highest educational level. Multilevel regression analyses were conducted to examine the associations of the parental and adolescents’ perceptions of traffic safety and crime safety with IM. The perceptions of traffic safety and crime safety were included as independent variables, and both IM and AIM were alternately entered as dependent variables. Again, these analyses were controlled for adolescents’ gender and parents’ highest educational level. The analyses were performed using the Statistical Package for Social Sciences (SPSS Version 20.0 for Windows, IBM Corp., Armonk, NY, USA), and the level of significance was set at p<0.05.

## Results

### Descriptive statistics

The descriptive data of the participants are presented in Table 2. The parents’ mean age was 41.4 ± 8.9 years old, mainly mothers (76.5%) completed the parental questionnaire, and 40% of them had a college or university degree. The adolescents were mostly girls (54.7%) and their mean age was 13.2 ± 1.0 years old. No differences in socio-demographic characteristics were found between boys and girls. We also found no gender differences for overall IM and AIM, but boys were allowed to travel further by bike than girls (p = 0.03).

**Table 2:** Descriptive data of the participants.

	All n = 243	Boys n = 110 46.3%	Girls n = 133 54.7%	*p*
Parents’ gender (n(%))				
Father	57 (23.5)	29 (26.4)	28 (21.1)	0.33
Mother	186 (76.5)	81 (73.6)	105 (78.9)	
Parents’ age (M± SD)	41.4±8.9	41.6±8.6	41.3±9.1	0.75
Parents’ highest educational level (n(%))				
Primary or secondary school	141 (60)	63 (59.4)	78 (60.5)	0.87
College or University	94 (40)	43 (40.6)	51 (39.5)	
Parents’ BMI (M± SD)	24.3±4.4	24.1±4.5	24.6±4.4	0.41
Adolescents’ age (M± SD)	13.2 ±1.0	13.1±1.0	13.2±1.0	0.56
Adolescents’ BMI (M± SD)	18.8 ±3.1	18.4±3.3	19.2±3.0	0.06
IM minutes across mode of transport [Med (Q1-Q3)]				
Walking	5.0 (0–20.0)	5.0 (0–20.0)	10.0 (0–15.5)	0.46
Cycling	**10.0(1.0–20.0)**	**10.0 (5.0–20.0)**	**10.0 (0–15.5)**	**0.03**
Public transport	6.0 (0–20.0)	0 (5.0–20.0)	10.0 (0–20.0)	0.63
IM* [Med (Q1-Q3)]	30.0 (10.0–51.2)	30.0 (10.0–55.0)	30.0 (15.0–50.0)	0.38
AIM** [Med (Q1-Q3)]	20.0 (8.7–40.0)	20.0 (7.0–41.2)	20.0 (10.0–30.0)	0.20

M = mean, SD = standard deviation, Med = median, Q1 = quartile 1, Q3 = quartile 3,

*IM = mean of the time walking, cycling, and using public transport without adult accompaniment,

**AIM = mean of the time walking and cycling without adult accompaniment

The means of the parents’ and adolescents’ perceptions of traffic safety and crime-related safety are presented in Table 3.

**Table 3:** Descriptive parents’ and adolescents’ perceptions of traffic safety and crime-related safety.

	Parents (M ± SD)	Adolescents (M ± SD)
**Traffic safety**		
Amount of traffic ^a^	2.3±0.9	2.2±0.8
Speed of traffic ^a^	2.9±0.8	3.0±0.8
Speeding ^a^	3.1±0.8	2.7±0.8
Exhaust fumes ^a^	2.6±0.8	2.2±0.8
Good lighting at night ^b^	2.7±0.7	3.1±0.7
Clear view of pedestrians ^b^	2.6±0.7	2.9±0.7
Presence of crosswalks and signals ^b^	2.6±09	2.9±0.9
Safety at crosswalks ^b^	2.6±0.8	3.0±0.7
**Crime safety**		
Crime ^a^	1.8±0.7	1.7±0.7
Crime at night ^a^	1.9±0.9	1.7±0.8
Hurt by a stranger (alone) ^a^	1.9±0.8	1.5±0.7
Hurt by a stranger (with friend) ^a^	1.7±0.8	1.3±0.6
Hurt by a stranger (walking) ^a^	1.8±0.8	1.4±0.7
Hurt by a stranger (nearby park) ^a^	2.1±0.9	1.7±0.8

^a^ higher scores reflect a less safe environment

^b^ higher scores reflect a safer environment

M = mean, SD = standard deviation

### Relation between parents’ and adolescents’ perception of traffic safety and crime-related safety

The results of the regression analyses are presented in Table 4. All the perceptions of traffic safety and crime-related safety differed between parents and adolescents, except for the amount of traffic and speed of traffic. Regarding traffic, parents had a higher perception of speeding and of exhaust fumes than adolescents (both p<0.001). Adolescents, however, reported more presence of good lighting at night, that pedestrians were visible from the houses nearby, a higher presence of crossings and signals for pedestrians, and more safety when crossing the streets than parents (all p<0.001). Concerning crime-related safety, parents reported more crime in the neighborhood and more fear of being hurt by a stranger (when commuting alone in the neighborhood, commuting with a friend, walking through the neighborhood and being in a nearby park), than adolescents (all p<0.001 except for crime p = 0.035). Furthermore, parents rather perceived that walking at night was not safe because of crime (p = 0.001) compared to adolescents.

**Table 4:** Multilevel regression analyses between age groups and perceptions of traffic safety and crime-related safety.

Dependent variable	β	SE	95% CI	
			Lower	Upper
**Traffic safety**				
Amount of traffic	-0.14	0.08	-0.29	0.16
Speed of traffic	0.07	0.08	-0.08	0.22
Speeding	**-0.42**	**0.07**	**-0.57**	-0.28**
Exhaust fumes	**-0.33**	**0.08**	**-0.48**	-0.18**
Good lighting at night	**0.37**	**0.06**	**0.24**	0.51**
Clear view of pedestrians	**0.33**	**0.06**	**0.20**	0.47**
Presence of crosswalks and signals	**0.33**	**0.08**	**0.17**	0.49**
Safety at crosswalks	**0.36**	**0.07**	**0.22**	0.50**
**Crime safety**				
Crime	**-0.14**	**0.6**	**-0.27**	-0.01*
Crime at night	**-0.26**	**0.78**	**-0.41**	-0.10**
Hurt by a stranger (alone)	**-0.35**	**0.07**	**-0.50**	-0.20**
Hurt by a stranger (with friend)	**-0.41**	**0.65**	**-0.54**	-0.29**
Hurt by a stranger (walking)	**-0.42**	**0.07**	**-0.56**	-0.29**
Hurt by a stranger (nearby park)	**-0.36**	**0.08**	**-0.51**	-0.20**

CI = confidence interval;

*p<0.05;

**p<0.01

### Associations of parents’ and adolescents’ perceptions of traffic- and crime-related safety with IM

The associations of both parents’ and adolescents’ perceptions of traffic safety and crime-related safety with IM and AIM are shown in Table 5. Several parental perceptions were associated with IM and AIM, but none of the adolescent perceptions were associated with IM or AIM.

**Table 5:** Multilevel regression analyses between parental and adolescents’ perception of traffic safety and crime-related safety with independent mobility and active independent mobility.

	Independent mobility			Active Independent Mobility		
	β	SE	CI (95%)	β	SE	CI (95%)
**Traffic safety**						
**Adolescents’ perception**						
Amount of traffic	-5.10	9.20	-23.25, 13.06	-1.54	6.51	-14.38, 11.30
Speed of traffic	10.88	8.36	-5.61, 27.38	7.46	5.88	-4.15, 19.07
Speeding	-5.23	8.16	-21.33, 10.88	-4.68	5.79	-16.11, 6.74
Exhaust fumes	6.36	9.33	-12.06, 24.78	-1.50	6.66	-14.64, 11.65
Good lighting at night	-17.89	9.62	-36.89, 1.11	-12.54	6.85	-26.06, 0.98
Clear view of pedestrian	0.92	9.33	-17.50, 19.35	3.59	6.60	-9.42, 16.61
Presence of crosswalks and signals	-2.00	7.87	-17.55, 13.54	-2.87	5.47	-13.66, 7.93
Safety at crosswalks	12.36	10.27	-7.92, 32.63	8.01	7.36	-6.51, 22.53
**Parents’ perception**						
Amount of traffic	-12.35	8.69	-29.51, 4.81	-7.40	6.34	-19.91, 5.11
Speed of traffic	3.94	7.46	-10.78, 18.67	6.47	5.38	-4.14, 17.08
Seeding	-7.11	8.58	-24.04, 9.82	-2.50	6.24	-14.83, 9.82
Exhaust fumes	-14.17	8.33	-30.61, 2.27	-12.58*	**6.02**	**-24.45, -0.70**
Good lighting at night	11.69	9.15	-6.37, 29.76	8.80	6.61	-4.24, 21.85
Clear view of pedestrian	-18.58*	**9.21**	**-36.76, -0.41**	-10.91	6.66	-24.07, 2.24
Presence of crosswalks and signals	-13.91	7.42	-28.56, 0.74	-16.32*	**5.37**	**-26.92, -5.73**
Safety at crosswalks	14.21	9.01	-3.57, 31.99	15.75*	**6.53**	**2.85, 28.64**
**Crime safety**						
**Adolescents’ perception**						
Crime	-6.42	11.36	-28.84, 16.00	-5.84	8.02	-21.68, 10.00
Crime at night	10.56	11.65	-12.43, 33.55	9.05	7.74	-6.22, 24.33
Hurt by a stranger (alone)	-15.19	15.02	-44.84, 14.46	-11.39	10.74	-32.59, 9.81
Hurt by a stranger (with friend)	0.83	21.42	-41.46, 43.12	14.47	15.25	-15.62, 44.57
Hurt by a stranger (walking)	26.73	18.96	-10.71, 64.17	11.75	13.54	-14.96, 38.47
Hurt by a stranger (nearby park)	-0.99	10.11	-20.95, 18.96	-4.79	7.22	-19.04, 9.47
**Parents’ perception**						
Crime	11.66	11.68	-11.40, 34.72	4.17	8.58	-12.77, 21.12
Crime at night	-6.73	10.84	-28.13, 14.66	-2.96	7.96	-18.67, 12.75
Hurt by a stranger (alone)	37.59*	**14.23**	**9.50, 65.68**	22.02*	**10.19**	**1.91, 42.12**
Hurt by a stranger (with friend)	-35.38*	**15.58**	**-66.15, -4.61**	-25.42*	**11.47**	**-48.06, -2.78**
Hurt by a stranger (walking)	8.13	18.10	-27.60, 43.86	13.21	13.20	-12.85, 39.27
Hurt by a stranger (nearby park)	-7.37	10.66	-28.42, 13.68	-4.60	7.88	-20.15, 10.95

CI = confidence interval;

*p<0.05;

More parental concerns about being hurt by a stranger when the adolescent is with friends and having clear view of pedestrians from houses nearby were associated with lower IM. When parents reported more concerns about being hurt by a stranger when the adolescent was alone, higher IM was reported.

Concerning AIM, parental perceptions of more safety at crosswalks and less presence of exhaust fumes from the cars were associated with higher AIM, and when parents reported more concerns about the adolescent being hurt when being with a friend, AIM was lower. Again, more parental concern about being hurt by a stranger when the adolescent is alone was associated with higher AIM and a higher presence of crosswalks and signals to cross busy roads was associated with lower AIM.

## Discussion

The aims of this study were (i) to analyze the differences in perceptions of traffic- and crime-related safety in the same neighborhood environment between adolescents and parents, and (ii) to analyze the associations of adolescents’ and parents’ perceptions with IM and AIM. The main results showed that parents and adolescents have different traffic and crime-related safety perceptions in the same environment, and only some parental perceptions, but not adolescents’, were associated with IM and AIM.

The perceptions of traffic safety and crime-related safety in the same environment were different between adolescents and parents, except for the amount and the speed of traffic. Parents showed more concerns about traffic safety and crime-related safety, while adolescents were more confident about both of them. This result is consistent with other studies comparing perceptions of the environment between parents and children in relation to active commuting to school. These studies indicated that parents reported almost twice more presence of traffic and stranger danger than both children and adolescents [45,46]. In fact, children reported that parents were more worried than them about traffic and crime safety [45]. Nevertheless, fear of traffic may not be unfounded, since 44% of the injury-related mortality in youth in developed countries is caused by transport accidents [47]. Belgium is the most congested country in Europe in terms of hours spent in traffic [48] and, during the past years, traffic jams have more than doubled. In this sense, parents are probably more aware than adolescents of the actual situation, and they probably have a deeper background resulting in more negative perceptions of traffic safety. A strategy to increase the parental perceptions of traffic safety may be to develop educational programs for both parents and their children, including activities where they commute together in order to increase parents’ confidence in their child’s commuting behavior [49] and improve their perceptions of the neighborhood.

Our findings showed that the adolescents’ perceptions of traffic and crime-related safety were not associated with IM or AIM. To date, no previous studies have examined the association of these factors with IM, but some studies have investigated the link with the active commuting behavior. A study in US children (10–12 years old) indicated that only children’s perceived presence of parks for children was associated with active mobility [45], and a Swedish study showed that adolescent-reported fear of traffic and crime was related to active commuting (47).

In the present study, the parental perception of traffic, particularly crosswalks and exhaust fumes, was associated with IM and AIM, but our findings were contradictory. Perceiving more safety at crosswalks, less exhaust fumes, and less presence of crossing signals were associated with higher AIM, whereas a clearer view of pedestrians from nearby houses was associated with a lower IM. The perception of traffic is one of the most important barriers for parents in relation to active commuting [50], and it has been associated with lower rates of active commuting in USA adolescents [51]. In fact, in the current study more traffic safety items were related to AIM than to overall IM. In relation to parental perception of traffic safety, a study conducted in Belgian children (10–12 years old) found that parental-perceived traffic safety was positively associated with AIM (for cycling) [25]. The presence of cars and air pollution have become a problem in Belgium because of the congestion of traffic [48], and further policy actions to develop programs focusing on reducing car emission and the amount of cars will be important to solve this public health problem. In addition, when there was a clear view of the adolescent from the houses nearby, IM was lower. This result might be explained by the current perceived erosion of trust and social relations in many communities in the last years [52], which may cause less faith in the good intentions of the community nearby.

In relation to crime-related safety, the parental perception of fear of the adolescent being hurt by a stranger when they were with friends was associated with a lower IM, whereas the parental fear of the adolescent being hurt when they were alone was associated with higher IM. In this regard, Australian children (10–12 years old) of parents with more fear of strangers had limited IM, and this association differed between boys and girls [53]. Consequently, strategies that help parents to recognize fear and its consequences are important to increase IM. The mixed results about stranger danger might be related to the assumption that adolescents take more risks when they are accompanied by friends due to their peers’ influences in comparison to when they are alone [54]. However, it is only a speculation and further qualitative research with focus groups could offer a deeper insight into the reasons of this counterintuitive result. Furthermore, it is important to improve the community and social relations in order to increase IM and AIM, since social networks are key to achieve higher levels of independence [55]. This is also crucial to rise active commuting rates, because the fear of crime is higher when there is less pedestrian movement [56].

In addition, active commuting is related to IM [57]. Thus, strategies to increase the active commuting to school and other destinations may be important to increase adolescents’ IM. A high variety of interventions focusing on increasing active commuting rates in children and adolescents have been conducted worldwide [58,59], specifically though interventions on school promotion more than policy and environmental changes [60]. Unfortunately, the interventions conducted did not achieve a high increase of active travels [61]. Few interventions targeted secondary school students and they focussed mostly on educational changes [62]. Thus, it is important to continue implementing new global interventions (i.e. environmental, social, personal) to promote active commuting and IM in children and adolescents.

The advantages of this study are the use of a large sample of paired adolescents and parents from different neighborhoods and the fact that traffic safety and crime-related safety were assessed using a validated questionnaire. Nevertheless, some limitations of this study should be acknowledged. First, since the study was executed in only one city of Belgium, generalizability of the findings is limited. Second, a cross-sectional design was used, which precludes any assumptions about causality. Lastly, we measured IM using the average minutes that participants commuted independently. Since information about the total mobility (i.e. minutes/week or distance) is lacking, we do not know how much of the participants’ total mobility is independent. In future research, it would be interesting to include a more detailed assessment of IM and to test the longitudinal changes in adolescents’ and parents’ perceptions across different geographical contexts. In addition, according to the ecological model explained before [23] it would be interesting to assess personal correlates as motivation or behavioral patterns to build a more complete model of the independent mobility.

In conclusion, parents and adolescents living in the same environment had different perceptions of traffic and crime-related safety, and only some parental perceptions, but not adolescents’ perceptions, were associated with IM and AIM. Future urban policy efforts should address environments where parents perceive sufficient levels of safety to increase the levels of IM in adolescents. In addition, educational programs focusing on improving parental perceptions of traffic- and crime-related safety and on the importance of reducing car use will be required to increase adolescents’ IM and AIM.

## Supporting information

S1 File Database. (SAV)

## References

[1] WHO. Global recommendations on physical activity for health [Internet]. Geneva; 2010 http://www.who.int/dietphysicalactivity/publications/9789241599979/en/26180873

[2] Biddle SJH, Asare M. Physical activity and mental health in children and adolescents: a review of reviews. Br J Sports Med. England; 2011;45: 886–895. 10.1136/bjsports-2011-090185 21807669

[3] Bunketorp Kall L, Malmgren H, Olsson E, Linden T, Nilsson M. Effects of a Curricular Physical Activity Intervention on Children ‘ s School Performance, Wellness, and Brain Development * ABSTRACT. J Sch Health. United States; 2015;85: 704–713. 10.1111/josh.12303 26331753

[4] Hsieh P-L, Chen M-L, Huang C-M, Chen W-C, Li C-H, Chang L-C. Physical activity, body mass index, and cardiorespiratory fitness among school children in Taiwan: a cross-sectional study. Int J Environ Res Public Health. Switzerland; 2014;11: 7275–7285. 10.3390/ijerph110707275 25032742 PMC4113875

[5] Janssen I, Leblanc AG. Systematic review of the health benefits of physical activity and fitness in school-aged children and youth. Int J Behav Nutr Phys Act. England; 2010;7: 1–16. 10.1186/1479-5868-7-40 20459784 PMC2885312

[6] Armstrong N. Young people are fit and active—Fact or fiction? J Sport Heal Sci. Elsevier Ltd; 2012;1: 131–140. 10.1016/j.jshs.2012.05.003

[7] Telama R, Yang X, Viikari J, Valimaki I, Wanne O, Raitakari O. Physical activity from childhood to adulthood: a 21-year tracking study. Am J Prev Med. Netherlands; 2005;28: 267–273. 10.1016/j.amepre.2004.12.003 15766614

[8] Sallis JF, Cervero RB, Ascher W, Henderson KA, Kraft MK, Kerr J. An Ecological Approach to Creating Active Living Communities. Annu Rev Public Heal. 2006;27: 297–322. 10.1146/annurev.publhealth.27.021405.102100 16533119

[9] De Meester F, Van Dyck D, De Bourdeaudhuij I, Cardon G. Parental perceived neighborhood attributes: associations with active transport and physical activity among 10–12 year old children and the mediating role of independent mobility. BMC Public Health. England; 2014;14: 631 10.1186/1471-2458-14-631 24950713 PMC4229936

[10] Carver A, Timperio AF, Hesketh KD, Ridgers ND, Salmon JL, Crawford DA. How is active transport associated with children’s and adolescents’ physical activity over time? Int J Behav Nutr Phys Act. BioMed Central Ltd; 2011;8: 126 10.1186/1479-5868-8-126 22081977 PMC3226569

[11] Tudor-Locke C, Ainsworth BE, Popkin B. Active commuting to school: An overlooked source of childrens’ physical activity? Sport Med. 2001;31 10.2165/00007256-200131050-00001 11347681

[12] Chillón P, Hales D, Vaughn A, Gizlice Z, Ni A, Ward DS. A cross-sectional study of demographic, environmental and parental barriers to active school travel among children in the United States. Int J Behav Nutr Phys Act. 2014;11: 61 10.1186/1479-5868-11-61 24885862 PMC4032634

[13] Lubans DR, Boreham CA, Kelly P, Foster CE. The relationship between active travel to school and health-related fitness in children and adolescents: a systematic review. 2011; 10.1186/1479-5868-8-5 PMC3039551 21269514

[14] Whitzman C, Mizrachi D. Vertical living kids: Creating supportive high rise environments for children in Melbourne, Australia. Melbourne: The University of Melbourne; 2009.

[15] Schoeppe S, Duncan MJ, Badland HM, Oliver M, Browne M. Associations between children’s independent mobility and physical activity. BMC Public Health. England; 2014;14: 91 10.1186/1471-2458-14-91 24476363 PMC3932047

[16] Garcia-cervantes L, Haese SD, Izquierdo-Gomez R, Padilla-Moledo C, Fernandez-Santos JR, Cardon G, et al Physical Activity Coparticipation and Independent Mobility as Correlates of Objectively Measured Nonschool Physical Activity in Different School Grades. The UP&DOWN Study. J Phys Act Health. United States; 2016;13: 747–753. 10.1123/jpah.2015-0415 26900750

[17] Hillman M, Adams J, Whitelegg J. One false move … a study of children’s independent mobility. London: Policy Studies Institute; 1990.

[18] Prezza M, Pacilli MG. Current fear of crime, sense of community, and loneliness in italian adolescents: The role of autonomous mobility and play during childhood. J Community Psychol. Wiley Subscription Services, Inc., A Wiley Company; 2007;35: 151–170. 10.1002/jcop.20140

[19] Carver A, Panter JR, Jones AP, van Sluijs EMF, Van Sluijs EMF. Independent mobility on the journey to school: A joint cross-sectional and prospective exploration of social and physical environmental influences. J Transp Heal. Netherlands: Elsevier; 2014;1: 25–32. 10.1016/j.jth.2013.12.003 25568839 PMC4278439

[20] Somerville LH, Casey BJ. Developmental neurobiology of cognitive control and motivational systems. Curr Opin Neurobiol. 2010;20: 236–241. 10.1016/j.conb.2010.01.006 20167473 PMC3014528

[21] Shaw B, Watson B, Frauendienst B, Redecker A, Jones T, Hillman M. Children’s independent mobility: a comparative study in England and Germany (1971–2010). London: Policy Studies Institute; 2013.

[22] Fyhri A, Hjorthol R, Mackett RL, Fotel TN, Kyttä M. Children’s active travel and independent mobility in four countries: Development, social contributing trends and measures. Transp Policy. 2011;18: 703–710. 10.1016/j.tranpol.2011.01.005

[23] Mandic S, La Barra SLD, Bengoechea EG, Stevens E, Moore A, Middlemiss M, et al Personal, social and environmental correlates of active transport to school among adolescents in Otago, New Zealand. J Sci Med Sport. Sports Medicine Australia; 2015;18: 432–437. 10.1016/j.jsams.2014.06.012 25027770

[24] Carver A, Timperio AF, Crawford DA. Young and free? A study of independent mobility among urban and rural dwelling Australian children. J Sci Med Sport. Sports Medicine Australia; 2012;15: 505–510. 10.1016/j.jsams.2012.03.005 22497720

[25] Ghekiere A, Deforche B, Carver A, Mertens L, De Geus B, Clarys P, et al Insights into children ‘ s independent mobility for transportation cycling—Which socio-ecological factors matter ? J Sci Med Sport. Sports Medicine Australia; 2017;20: 267–272. 10.1016/j.jsams.2016.08.002 27566898

[26] Stone MR, Faulkner GE, Mitra R, Buliung RN. The freedom to explore: examining the influence of independent mobility on weekday, weekend and after-school physical activity behaviour in children living in urban and inner-suburban neighbourhoods of varying socioeconomic status. Int J Behav Nutr Phys Act. England; 2014;11: 5 10.1186/1479-5868-11-5 24450739 PMC3902417

[27] Seabrook JA, Avison WR. Socioeconomic status and cumulative disadvantage processes across the life course: implications for health outcomes. Can Rev Sociol. United States; 2012;49: 50–68. 10.1111/j.1755-618x.2011.01280.x 22586837

[28] Witten K, Kearns R, Carroll P, Tava N. Children’s Geographies New Zealand parents’ understandings of the intergenerational decline in children’s independent outdoor play and active travel. Child Geogr. 2013;11: 37–41.

[29] Wolfe MK, McDonald NC. Association Between Neighborhood Social Environment and Children’s Independent Mobility. J Phys Act Heal. Human Kinetics; 2016;13: 970–979. 10.1123/jpah.2015-0662 27171119

[30] Mammen G, Faulkner G, Buliung R, Lay J. Understanding the drive to escort: a cross-sectional analysis examining parental attitudes towards children’s school travel and independent mobility. BMC Public Health. BMC Public Health; 2012;12: 862 10.1186/1471-2458-12-862 23051005 PMC3534151

[31] Carver A, Timperio A, Crawford D. Playing it safe: The influence of neighbourhood safety on children’s physical activity—A review. Health Place. 2008;14: 217–227. 10.1016/j.healthplace.2007.06.004 17662638

[32] Giles-Corti B, Kelty SF, Zubrick SR, Villanueva KP. Encouraging Walking for Transport and Physical Activity in Children and Adolescents. Sport Med. 2009;39: 995–1009. 10.2165/11319620-000000000-00000 19902982

[33] Ahern S. Individual and structural influences on parent’s transport choices for the school run: a qualitative interview study. UK Society for Behavioural Medicine 11th Annual Scientific Meeting Biology, Behaviour & Environment. Newcastle; 2015.

[34] Janssen I. Crime and perceptions of safety in the home neighborhood are independently associated with physical activity among 11-15year olds. Prev Med (Baltim). Elsevier B.V.; 2014;66: 113–117. 10.1016/j.ypmed.2014.06.016 24963893

[35] Carver A, Veitch J, Sahlqvist S, Crawford D, Hume C. Active transport, independent mobility and territorial range among children residing in disadvantaged areas. J Transp Heal. Elsevier; 2014;1: 267–273. 10.1016/j.jth.2014.01.004

[36] Panter JR, Jones AP, van Sluijs EMF. Environmental determinants of active travel in youth: A review and framework for future research. Int J Behav Nutr Phys Act. 2008;5: 1–14. 18573196 10.1186/1479-5868-5-34 PMC2483993

[37] Defoe IN, Dubas JS, Figner B, van Aken MAG. A meta-analysis on age differences in risky decision making: Adolescents versus children and adults. Psychological Bulletin. Defoe Ivy N.: Department of Developmental Psychology, Utrecht University, Utrecht, Netherlands, 3508 TC, i.n.defoe@uu.nl: American Psychological Association; 2015 pp. 48–84. 10.1037/a0038088 25365761

[38] Valentine G. “My Son’s a Bit Dizzy.” “My Wife’s a Bit Soft”: Gender, children and cultures of parenting. Gender, Place Cult. Routledge; 1997;4: 37–62. 10.1080/09663699725495

[39] Carver A, Salmon J, Campbell K, Baur L, Garnett S, Crawford D. How do perceptions of local neighbourhood relate to adolescent’s walking and cycling? Am J Heal Promot. 2005;20 10.4278/0890-1171-20.2.139 16295706

[40] Schoeppe S, Duncan MJ, Badland HM, Alley S, Williams S, Rebar AL. Socio-demographic factors and neighbourhood social cohesion influence adults ‘ willingness to grant children greater independent mobility : A cross-sectional study. BMC Public Health. BMC Public Health; 2015; 1–8. 26198083 10.1186/s12889-015-2053-2 PMC4509769

[41] Bates B, Stone MR. Measures of outdoor play and independent mobility in children and youth: A methodological review. J Sci Med Sport. Sports Medicine Australia; 2015;18: 545–552. 10.1016/j.jsams.2014.07.006 25128461

[42] Mackett R, Brown B, Gong YI, Kitazawa KAY, Paskins J. Children’s Independent Movement in the Local Environment. Buit Environ. 2005;33: 454–468.

[43] D’Haese S, Van Dyck D, De Bourdeaudhuij I, Deforche B, Cardon G. The association between objective walkability, neighborhood socio-economic status, and physical activity in Belgian children. Int J Behav Nutr Phys Act. England; 2014;11: 104 10.1186/s12966-014-0104-1 25148724 PMC4243938

[44] Rosenberg D, Ding D, Sallis JF, Kerr J, Norman GJ, Durant N, et al Neighborhood Environment Walkability Scale for Youth (NEWS-Y): Reliability and relationship with physical activity. Prev Med (Baltim). Elsevier Inc.; 2009;49: 213–218. 10.1016/j.ypmed.2009.07.011 19632263

[45] Timperio A, Crawford D, Telford A, Salmon J. Perceptions about the local neighbourhood and walking and cycling among children. Prev Med (Baltim). 2004;38 10.1016/j.ypmed.2003.09.026 14672640

[46] Forman H, Kerr J, Norman GJ, Saelens BE, Durant NH, Harris SK, et al Reliability and validity of destination-specific barriers to walking and cycling for youth. Prev Med (Baltim). United States; 2008;46: 311–316. 10.1016/j.ypmed.2007.12.006 18206220

[47] UNICEF. Deaths by injury in rich Nations [Internet]. Innocenti report card. Florence; 2001. 88-85401-71-6

[48] European Commision Staff. Country report belgium 2017. Communication from the commission to the european parliament, the council, the european central bank and the eurogroup. Brussels; 2017.

[49] Ghekiere A, Carver A, Veitch J, Salmon J, Deforche B, Timperio A. Does parental accompaniment when walking or cycling moderate the association between physical neighbourhood environment and active transport among 10–12 year olds? J Sci Med Sport. Sports Medicine Australia; 2016;19: 149–153. 10.1016/j.jsams.2015.01.003 25661722

[50] Huertas-Delgado FJ, Herrador-Colmenero M, Villa-González E, Aranda-Balboa MJ, Cáceres V, Mandic S, et al Parental Barriers to Active Commuting to School in Spanish Children and Adolescents. Eur J Public Health. 2017;27: 416–421. 28108594 10.1093/eurpub/ckw249

[51] Oluyomi AO, Lee C, Nehme E, Dowdy D, Ory MG, Hoelscher DM. Parental safety concerns and active school commute: correlates across multiple domains in the home-to-school journey. Int J Behav Nutr Phys Act. International Journal of Behavioral Nutrition and Physical Activity; 2014;11: 32 10.1186/1479-5868-11-32 24602213 PMC3975836

[52] Putnam RD. Bowling Alone: America’s Declining Social Capital. J Democr. 1995;6: 65–78.

[53] Foster S, Villanueva K, Wood L, Christian H, Giles-Corti B. The impact of parents’ fear of strangers and perceptions of informal social control on children’s independent mobility. Heal Place. Elsevier; 2014;26: 60–68. 10.1016/j.healthplace.2013.11.006 24374289

[54] Brechwald WA, Prinstein MJ. Beyond Homophily: A Decade of Advances in Understanding Peer Influence Processes. J Res Adolesc. 2011;21: 166–179. 10.1111/j.1532-7795.2010.00721.x 23730122 PMC3666937

[55] Veitch J, Bagley S, Ball K, Salmon J. Where do children usually play? A qualitative study of parents’ perceptions of influences on children’s active free-play. Health Place. 2006;12: 383–393. 10.1016/j.healthplace.2005.02.009 16814197

[56] Foster S, Giles-Corti B, Knuiman M. Neighbourhood design and fear of crime: A social-ecological examination of the correlates of residents’ fear in new suburban housing developments. Heal Place. Elsevier; 2010;16: 1156–1165. 10.1016/j.healthplace.2010.07.007 20719555

[57] Larouche R, Barnes J, Tremblay MS. Too Far to Walk or Bike ? Can J Public Heal. 2013;104: 487–489. 10.17269/cjph.104.4122 PMC6973793 24495826

[58] Chillón P, Evenson KR, Vaughn A, Ward DS. A systematic review of interventions for promoting active transportation to school. Int J Behav Nutr Phys Act. BioMed Central Ltd; 2011;8: 10 10.1186/1479-5868-8-10 21320322 PMC3050785

[59] Villa-Gonzalez E, Barranco-Ruiz Y, Evenson KR, Chillon P. Systematic review of interventions for promoting active school transport. Prev Med (Baltim). United States; 2018;111: 115–134. 10.1016/j.ypmed.2018.02.010 29496615

[60] Pang B, Kubacki K, Rundle-Thiele S. Promoting active travel to school: a systematic review (2010–2016). BMC Public Health. England; 2017;17: 638 10.1186/s12889-017-4648-2 28779756 PMC5545094

[61] Villa-Gonzalez E, Ruiz JR, Ward DS, Chillon P, Villa-González E, Ruiz JR, et al Effectiveness of an active commuting school-based intervention at 6-month follow-up. Eur J Public Health. England; 2016;26: 272–276. 10.1093/eurpub/ckv208 26578663

[62] Larouche R, Mammen G, Rowe DA, Faulkner G. Effectiveness of active school transport interventions: a systematic review and update. BMC Public Health. England; 2018;18: 206 10.1186/s12889-017-5005-1 29390988 PMC5796594

